# Impact of Lipid Composition on Membrane Partitioning and Permeability of Gas Molecules

**DOI:** 10.3390/membranes16010033

**Published:** 2026-01-04

**Authors:** Paween Mahinthichaichan, Ahmad Raeisi Najafi, Fraser J. Moss, Ardeschir Vahedi-Faridi, Walter F. Boron, Emad Tajkhorshid

**Affiliations:** 1 Theoretical and Computational Biophysics Group, NIH Resource for Macromolecular Modeling and Visualization, Beckman Institute for Advanced Science and Technology, Department of Biochemistry, and Center for Biophysics and Quantitative Biology, University of Illinois Urbana-Champaign, Urbana, IL 61801, USA; mahinth1@gmail.com (P.M.); arn55@drexel.edu (A.R.N.); 2 Department of Physiology and Biophysics, Case Western Reserve University, Cleveland, OH 44106, USA; fraser.moss@case.edu (F.J.M.); axv163@case.edu (A.V.-F.); wfb2@case.edu (W.F.B.)

**Keywords:** gas permeation, cellular membranes, molecular dynamics simulation

## Abstract

The permeation of different chemical substances across the membrane is of utmost importance to the life and health of a living cell. Depending on the nature of the permeant, the process is mediated by either the protein (e.g., membrane channels) or lipid phases of the membrane, or both. In the case of small and physiologically important gas molecules, namely O_2_ and CO_2_, the literature supports the involvement of both pathways in their transport. The extent of involvement of the lipid phase, however, is directly dependent on the nature of the lipid constituents of the membrane that determine its various structural and physicochemical properties. In this study, we use molecular dynamics simulation, as a method with sufficient spatial and temporal resolutions, to analyze these properties in heterogeneous lipid bilayers, composed of phospholipids with varied tails, sphingomyelin, and cholesterol, to different degrees. Together with the calculation of the free energy profiles, diffusion constants, and gas diffusivity, the results shed light on the importance of the lipid phase of membranes in gas transport rate and how they can be modulated by their lipid composition.

## 1. Introduction

Lipid membranes provide physical barriers that control the exchange and flow of substances in and out of living cells [1,2]. Unlike polar and charged molecules such as ions, which are energetically unfavorable to dissolve in the hydrophobic core of a lipid membrane [3,4], small, nonpolar gas species, such as O_2_, NO, and CO_2_, may diffuse readily through pure lipid bilayers. Although this mode has long been accepted as the main mechanism for gas transport across the cellular membranes  [5,6,7], some biological membranes exhibit surprisingly low gas permeabilities [8,9]. Examples include the gastric parietal chief cells [10], erythrocytes [11,12], and fiber cells of the eye lenses [13]. These reports evoke the question as to what degree the lipid composition can determine the gas permeability of a membrane. Distinct gas permeation rates have been reported for membranes composed of different lipid constituents [13,14,15,16,17,18,19,20]. Membrane channels, e.g., aquaporins and Rhesus (Rh) glycoproteins, are also documented to facilitate gas transport across the membrane [21,22,23,24,25,26,27,28], but their physiological significance in this regard remains disputed [29,30], mainly due to experimental limitations [31,32]. Demonstrating that the lipid composition can modulate gas permeability is the first step in establishing the physiological significance of membrane proteins in the overall gas exchange in living cells.

Given its microscopic results, molecular dynamics (MD) simulation can offer insights into the delivery and transport of molecules across the membranes at the atomic level. Physical properties, such as molecular partitioning and permeability coefficients can also be predicted using MD [3,33,34,35]. For years, lipid membranes have been simulated with only one glycerophospholipid type, such as POPC, DOPC, or POPE, which are highly fluid under ambient conditions. In recent years, all-atom simulations of membranes with heterogeneous lipid compositions containing multiple glycerophospholipid species, as well as cholesterol (CHL) and sphingomyelins (SM) have been performed [36,37,38].

**Figure 1:**
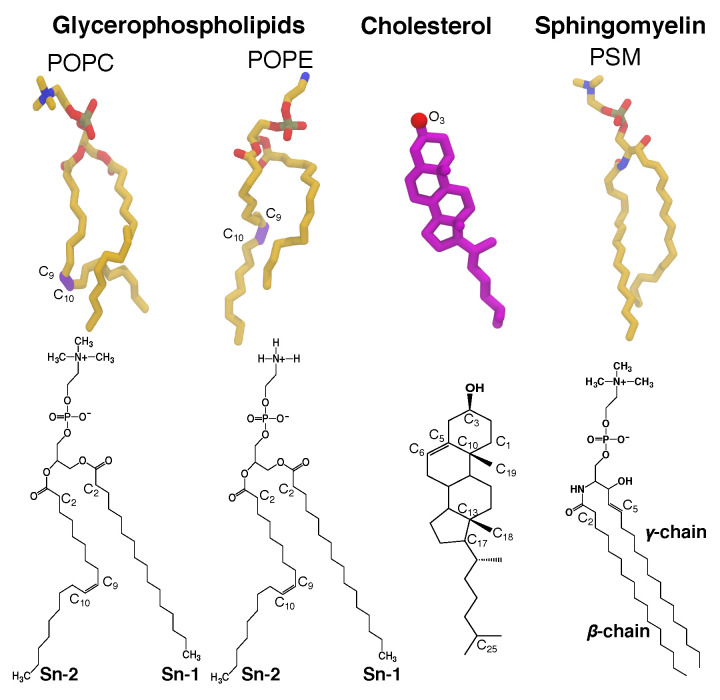
Representative lipids in biological membranes. Glycerophospholipids contain a diacylglycerol backbone attached to a phosphate head group. 1-palmitoyl-2-oleoyl-*sn*-glycero-3-phosphocholine (POPC) and 1-palmitoyl-2-oleoyl-*sn*-glycero-3-phosphoethanolamine (POPE) are among the common glycerophospholipids found in living cells. Cholesterol (CHL) is constituted of a planar steroid ring, decorated by two methyl groups at C_10_ and C_13_, a hydroxyl group at C_3_, and an 8-carbon aliphatic tail at C_17_. Sphingomyelin (SM), a type of sphingolipid found in animal cells, contains the phosphocholine head group attached to the sphingosine chain (γ-chain) and an amide-linked acyl chain (β-chain). Palmitoylsphingomyelin (PSM) is the most computationally and structurally studied sphingomyelin and has a similar size to a POPC. Its γ or sphingosine chain (1,3-dihydroxy-2-amino-4-octadecene) contains a *trans* double bond between C_4_ and C_5_, and a hydroxyl group attached to C_3_. It is the most common base in mammalian sphingomyelins. Its β or acyl chain is a saturated hydrocarbon tail composed of 16 carbon atoms. These structural features of SMs allow its interfacial region between the head group and the hydrophobic tails to act as both hydrogen bond donor and acceptor, whereas the one of glycerophospholipids can only act as hydrogen bond acceptor. Atomic images of POPC, POPE, CHL, and PSM lipids are shown in the **top panel**, and chemical structures are shown in the **bottom panel**.

CHL and SM (Figure 1) are key lipids for structural integrity and morphology of mammalian membranes, and perform other regulatory functions [2,39]. They are abundant in typical mammalian plasma membranes, including erythrocyte and lens membranes, which are reported to have low gas permeability [40]. In those membranes, CHL:phospholipid ratios can range from 1:1 to 4:1, where SM constitutes >15% of the lipid content [41,42].

CHL is enriched in the liquid-ordered phase of the membrane [43,44] and also known to induce lipid domain formations [45,46,47]. The clustering between SM with CHL has been implicated to the formation of liquid, highly-ordered domains, known as rafts, which have been proposed to be involved in cell signaling [48,49,50,51]. Other phospholipids, such as phosphotidylserine (PS), also play important roles in mammalian membranes. However, in this study, we focus on the impact of CHL and SM on the membrane, which are known to rigidify the membrane phase. The effects of CHL on membrane permeability have been probed by MD simulations. Previous studies with extensive simulations and analysis of membranes with high CHL content (>30 mole%) predicted a reduced water permeability [52,53]. Other studies also found apparent reduction in the partitioning of small molecules and gases in membranes with CHL relative to CHL-free membranes [54,55,56,57].

The present study examines the effects of lipid composition heterogeneity, particularly the presence of high CHL and SM, on gas permeability by explicitly simulating O_2_ or CO_2_ molecules in membranes. The sets of independent simulations comprise CHL-free (pure) bilayers of POPC, SM, or DPPC, binary compositions of POPC:CHL, SM:CHL, DPPC:CHL, POPC:SM, and POPC:DPPC, and ternary compositions of POPC:PSM:CHL and POPC:DPPC:CHL. SM is represented by the palmitoylsphingomyelin (PSM) (Figure 1). The fully saturated DPPC (dipalmitoylphosphatidylcholine) lipids account for the effects of lipid saturation on gas permeability. We explicitly calculated membrane partitioning free energy profiles and permeability coefficients of the gas molecules from the equilibrium MD trajectories. The results show clearly the dependence of the calculated gas permeability on the lipid composition; they demonstrate that high CHL content reduces the solubility of gas molecules in the membrane. Reduced gas permeability is pronounced in the simulated POPC-free PSM membranes, due to slow lipid dynamics and gel-liquid phase behavior under physiological body temperature (310 K).

## 2. Materials and Methods

### 2.1. Simulation Systems

The simulated membrane systems are summarized in Table 1. They comprise mixtures of POPC:PSM:CHL or POPC:DPPC:CHL lipids. The membrane patches were prepared using CHARMM-GUI [58]. The membranes were solvated with TIP3P water and ionized with 0.2 M NaCl. Structures and interactions of lipids (POPC, PSM, and CHL), water, ions, and gases were described by the CHARMM36 force field parameter set [59,60,61,62,63]. The initial dimension of each membrane plane was 100 Å × 100 Å.

Depending on the membrane system, a simulation was performed to equilibrate the system in the absence of gas molecules for tens to a few hundred nanoseconds. The area of the xy plane of each system was monitored, and the simulation was continued until it became steady. To explicitly describe the partitioning and permeation of gases, O_2_ or CO_2_, 125 copies of each species were added to the equilibrated systems. The molecules were initially placed in the aqueous solution. We refer to these types of simulations as “flooding” simulations.

### 2.2. Simulation Protocols

All simulations were performed using NAMD2 [64,65] with the CHARMM36 force field [66] and a time step of 2 fs. Force field parameters for O_2_ and CO_2_ are directly available in the CHARMM36 force field [66]. All of the simulations were performed under NPT ensemble with the flexible cell under the constant x/y ratio, with the *z* axis representing the membrane normal. The periodic boundary condition (PBC) was used throughout the simulations. To evaluate long-range electrostatic interactions in PBC without truncation, the particle mesh Ewald (PME) method [67] with a grid density of 1/Å^3^ was used. The temperature was maintained at 310 K by Langevin dynamics [68] with a damping coefficient γ of 1/ps. The modified Nosé–Hoover method [68,69], in which Langevin dynamics was used to maintain the pressure at 1 atm with a piston period of 200 fs. All bonds involving hydrogen atoms were kept rigid using the SHAKE algorithm [70]. The cutoff for van der Waals interactions was set at 12 Å.

### 2.3. Membrane Partitioning and Permeability of Gases

For the analysis, we focused on the last part of each trajectory, when the membrane was considered equilibrated with the gas molecules, initially placed in solution. In order to determine equilibration, we monitored the number of gas molecules in the membrane. Once this number plateaued, the system was considered equilibrated, and the rest of the trajectory was used for analysis.

The partitioning free energy profiles (ΔG) and membrane permeability coefficients ($p_{d,\mathrm{mem}}$) of O_2_ or CO_2_ were calculated using the last 175 ns part of trajectories for the 100% POPC membrane systems, and the last 400 ns parts of trajectories for the pure PSM, 0:50:50 POPC:PSM:CHL, and 0:50:50 POPC:DPPC:CHL systems. For the other membrane systems (50:0:50, 35:0:65, 50:50:0, 33:33:33, and 25:25:50 POPC:PSM:CHL, and 50:50:0 and 33:33:33 POPC:DPPC:CHL), which were simulated for 325 ns, we used the last 250 ns for the analysis. ΔG profiles representing the occupancy of the gas molecule was expressed as:

$$\Delta G=-RT\ln\frac{P_{i}}{P_{\mathrm{bulk}}},\;$$

with $P_{i}$ and $P_{\mathrm{bulk}}$ representing occupancy of the gas molecule at position *i* and in the aqueous solution, respectively. The VolMap plugin of VMD 1.9.2 [71] was used to calculate the occupancy coefficients, which were then projected onto a one-dimensional (*z*) axis (membrane normal) using the Volutil plugin.

$p_{d,\mathrm{mem}}$ was determined from the cumulative profiles of the number of permeation events (*N*) from either the extracellular side or the intracellular side over a time period (δt) using linear regression. It is in the unit of cm/s and expressed as:

$$p_{d,\mathrm{mem}}=\frac{m}{N_{A}C_{b}A},$$

where *m* is the slope of *N* over δt, $C_{b}$ is the concentration of the gas molecule in the aqueous solution, and *A* is the area of the xy plane of the membrane. $C_{b}$ was determined from the average number of the gas molecules outside the membrane, normalized by the number of water molecules in the same region.

The gas molecules were considered to be in the extracellular solution when they were at least 3 Å above the center of mass of the choline moiety of the lipid head groups, and within the membrane when they were at least 3 Å below the center of mass of the choline groups.

## 3. Results

### 3.1. Variation of Membrane Structure by Lipid Composition

#### 3.1.1. Occupancy Profiles

**Figure 2:**
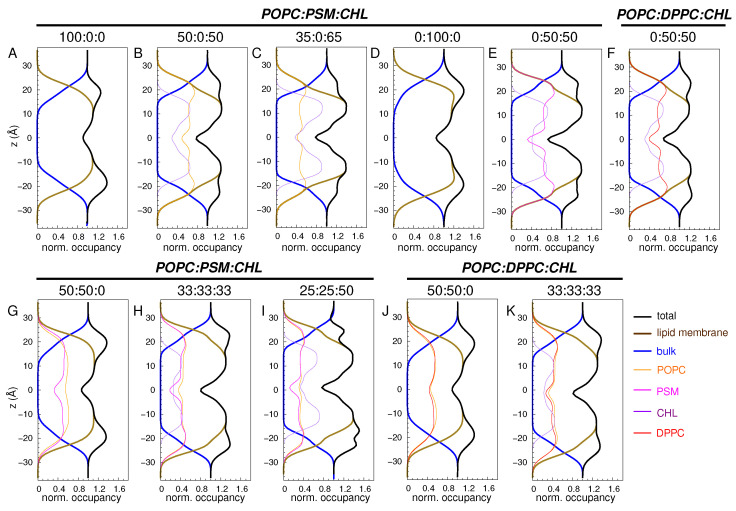
Atomic profiles of membrane constituents (lipids and water) plotted against the membrane normal (*z*) with z=0 indicating the membrane midplane. Water occupancy in the bulk was used to normalize the profiles. The intersection points of water and lipid profiles mark the water–lipid interfaces, where the phosphocholine head groups are located.

We first monitored the changes in membrane structure by calculating the atomic profiles of its constituents, including water and lipids (Figure 2).

The atomic densities or occupancies for individual components of the system were calculated by counting the number of atoms belonging to each component inside 1-Å slabs along the normal axis and normalized by the density of bulk water. In all the simulated membranes, the midpoint of the lipid bilayer (z=0) corresponds to the lowest occupancy region. In the pure POPC bilayer, as well as in the 50:50 POPC:PSM and POPC:DPPC bilayers (Figure 2A,G,J), the highest occupancy regions correspond to the head group regions located at z=±22 Å, inferring a membrane thickness of ∼44 Å. These points are at the intersection of the water and lipid occupancy profiles, marking the lipid-water interfaces.

Having simulated the POPC membranes with a high content of CHL (50 and 65 mole%) and without it, we found that CHL significantly alters the membrane profiles (Figure 2A vs. Figure 2B,C). In the presence of high CHL, the maximum density along the membrane normal spans from the head group regions to the lipid tails centered at z=±10 Å. CHL molecules are localized beneath the phospholipid head groups. Their positioning decreases the depth of water penetration into the membrane, as indicated by the observed dehydration between z=−15 and z=−10 Å, and between z=10 and z=15 Å.

For the pure PSM bilayer, the lipid-water interfaces center at z=±24 Å, thickening the membrane by ∼4 Å (Figure 2D). The membrane appears to be more condensed than the CHL-free POPC, POPC:PSM, and POPC:DPPC bilayers. Its average area-per-lipid is ∼51 Å^2^, whereas those of the CHL-free POPC, POPC:PSM, and POPC:DPPC bilayers are ∼65, 59, and 62 Å^2^, respectively. The maximum lipid density of the pure PSM bilayer plateaus to values between z=−20 and −5 Å, and between z=5 and 20 Å, respectively (Figure 2D). For the 50:50 PSM:CHL and DPPC:CHL bilayers, the distance between the two lipid-water interfaces increases to about z=50 Å. However, the PSM:CHL one is more condensed than the DPPC:CHL one by ∼15% (Figure 2E,F). Similar to the 50:50 and 35:65 POPC:CHL bilayers, a high CHL content decreases the hydration in both PSM:CHL and DPPC:CHL bilayers. This same behavior is also apparent in the 33:33:33 POPC:PSM:CHL and POPC:DPPC:CHL bilayers (Figure 2H,I,K).

#### 3.1.2. Lipid Ordering

**Figure 3:**
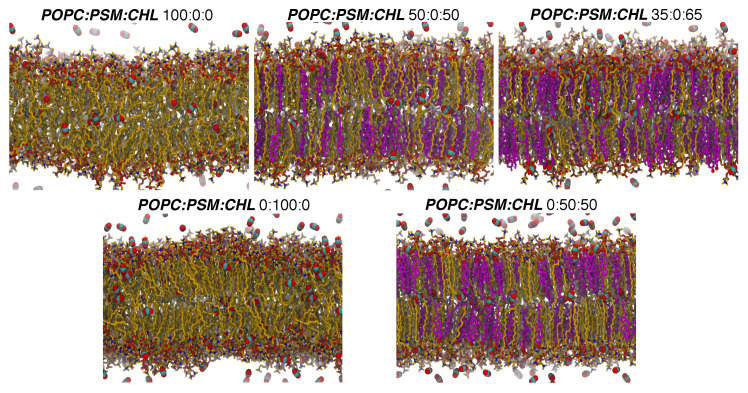
Membrane structures with different membrane lipid compositions and gas distributions. Membrane systems are labeled based on POPC:PSM:CHL or POPC:DPPC:CHL mole percentage. Lipid molecules are shown in the licorice representation. Oxygen atoms are shown as red balls. Carbon atoms of POPC and PSM lipids are shown in yellow, and those of CHL molecules are shown in purple. CO_2_ molecules, shown in space-filling with carbon atoms as cyan balls and oxygen atoms as red balls, are the representatives of gases in this figure.

The lipid molecules in the pure POPC bilayer, as well as those in the other CHL-free bilayers, appear disordered and highly fluid (Figure 3). Those in the simulated CHL-containing bilayers, on the other hand, are conformationally ordered and structurally extended. Uniquely, the pure PSM bilayer resembled more like a ripple phase (between gel and liquid phases). Its shape visibly appears undulated, and its lipid molecules appear clustered into small domains (Figure 3).

**Figure 4:**
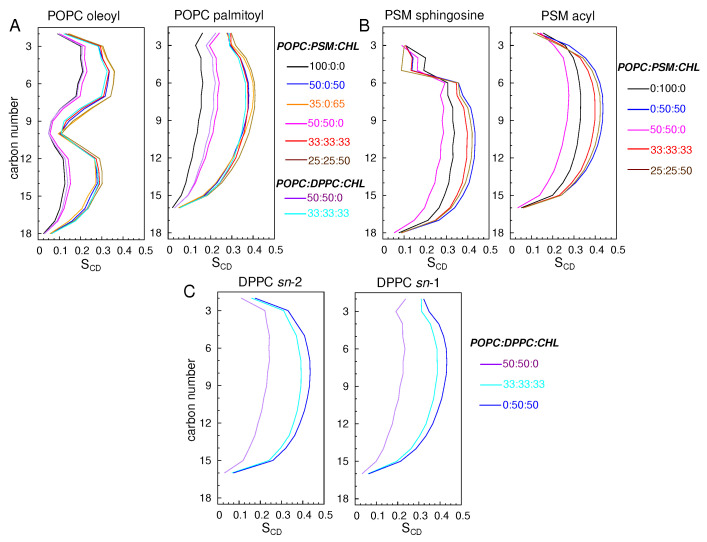
Deuterium order parameters ($S_{\mathrm{CD}}$) illustrating the disorder of POPC oleoyl and palmitoyl chains (**A**), PSM sphingosine and acyl chains (**B**), and DPPC *sn*-1 and *sn*-2 chains (**C**). $S_{\mathrm{CD}}=\left|1/2<3\cos^{2}\alpha -1>\right|$, where α is the angle between the membrane normal and a selected C-H bond vector. $S_{\mathrm{CD}}$ can also be calculated from quadrupolar splittings determined from ^2^H-NMR experiments [72,73,74,75,76]. Lipid chains are completely disordered or conformationally isotropic when $S_{\mathrm{CD}}=0$, and this is when they are oriented at the magic angle with respect to the magnetic field. The chains are perfectly in order or in the extended all-trans conformation when $S_{CD}$ = 0.5.

To quantify the conformational disorder of the lipid molecules, we calculated the deuterium order parameters ($S_{\mathrm{CD}}$) for the aliphatic chains of POPC and DPPC, the sphingosine and acyl chains of PSM (Figure 4).

An $S_{\mathrm{CD}}$ value of 0.5 indicates that the lipid molecules are ordered and that their tails are conformationally extended (not tilted). A value of 0 means that the lipids are highly disordered. Among the simulated membranes, the pure POPC bilayer is the most disordered membrane with the maximum $S_{\mathrm{CD}}$ values of ∼0.2 for both palmitoyl and oleoyl chains (Figure 4A). Lipids in the simulated CHL-containing bilayers are highly ordered with maximum $S_{\mathrm{CD}}$ values approaching 0.4 for POPC lipids and above 0.4 for PSM and DPPC lipids. In the pure PSM bilayer, $S_{\mathrm{CD}}$ values are slightly above ∼0.3, suggesting appreciable tilting of some lipid molecules, forming an undulated membrane. The lipid order is reduced in the 50:50 POPC:PSM bilayer with the drop of the maximum $S_{\mathrm{CD}}$ values of the PSM chains to 0.275 (Figure 4B). In the 50:50 POPC:DPPC bilayer, the $S_{\mathrm{CD}}$ values are ∼0.2 (Figure 4C).

**Figure 5:**
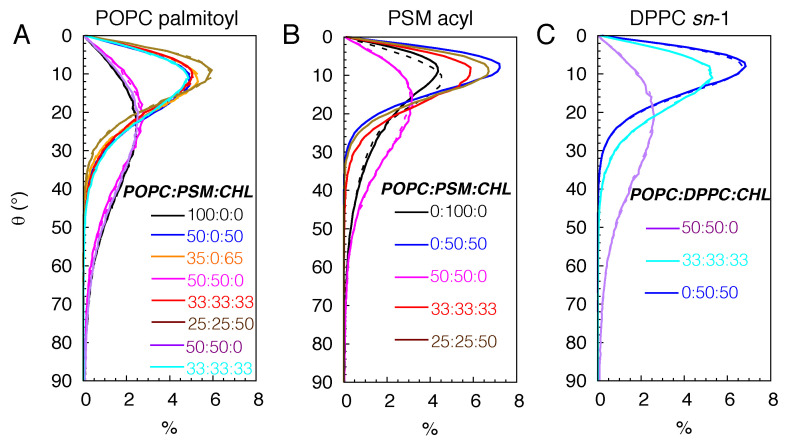
Tilting of fatty acid chains. θ is the angle between the vector connecting the C_2_ and C_16_ atoms and the membrane normal (0, 0, 1) for the upper leaflet and (0, 0, −1) for the lower leaflet. Solid lines correspond to the distributions in the upper leaflet, whereas dashed lines correspond to those in the lower leaflets.

To confirm the tilting of the lipid molecules, we also calculated the tilt angles (θ) of the lipid tails (using carbon atoms) with respect to the membrane normal. In the absence of CHL, lipid tails adopt a broad range of orientations with θ spanning from 0 to 90°; the distribution peaks are ∼25° for POPC and DPPC lipids and ∼20° for PSM lipids (Figure 5).

This reflects the conformational heterogeneity of the lipid molecules, showing some are highly tilted or structurally bent. In the simulated bilayers with CHL, lipid tails become significantly less tilted; the θ values range between 0 and 90° with the peaks at 10°, consistent with increasing lipid order. For the pure PSM bilayers, although the distribution peaks of θ are ∼10°, θ spreads from 0 to 70° (Figure 5B), confirming high degrees of tilting in some lipid molecules.

### 3.2. Membrane Partitioning and Permeability of Gas Molecules

#### 3.2.1. Pure POPC and Binary POPC:CHL Bilayers

**Figure 6:**
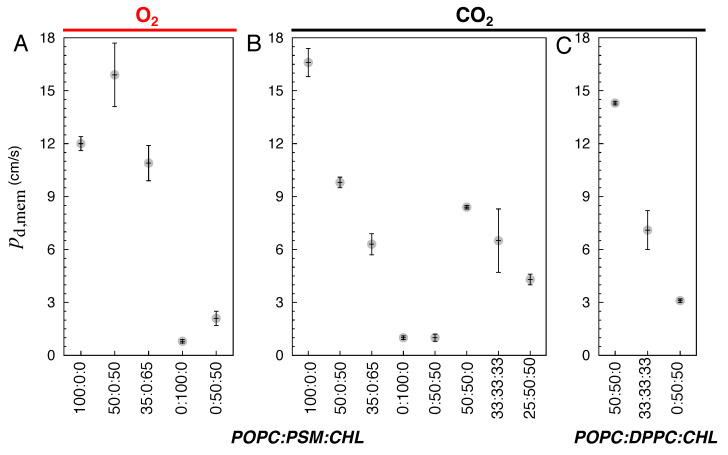
Calculated membrane permeability coefficients ($p_{d,\mathrm{mem}}$) of gas molecules: (**A**) O_2_ in POPC:PSM:CHL bilayers. (**B**) CO_2_ in POPC:PSM:CHL bilayers. (**C**) CO_2_ in POPC:DPPC:CHL bilayers.

**Figure 7:**
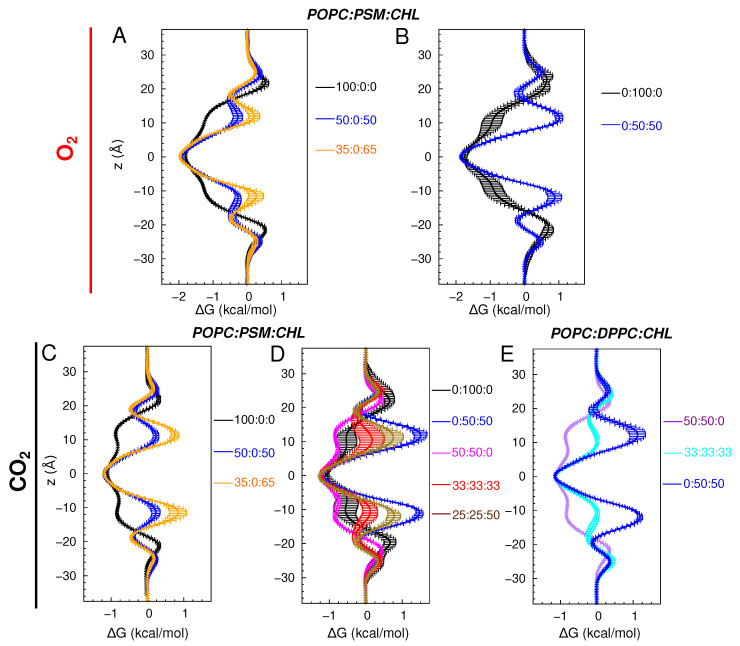
Partitioning free energy profiles (ΔG) of gas molecules plotted against the membrane normal (*z*) with z=0 indicating the membrane midplane, for (**A**,**B**) O_2_ in POPC:PSM:CHL bilayers. (**C**,**D**) CO_2_ in POPC:PSM:CHL bilayers. (**E**) CO_2_ in POPC:DPPC:CHL bilayers. The partitioning ΔG profiles of gases were calculated using the equilibrium portions of flooding simulation trajectories. Bars indicate standard deviation of the mean.

The changes in membrane structure and dynamics by lipid compositions can have profound effects on the membrane partitioning and permeability of gas molecules. Using the equilibrium flooding MD trajectories, we directly calculated the permeability coefficients ($p_{d,mem}$) and partitioning ΔG profiles of O_2_ and CO_2_ (Table 2, Figure 6 and Figure 7). For the pure POPC bilayers, the last 175 ns of the 225-ns simulations were used for the calculations. The calculated $p_{d,mem}$ values are ∼12 and 16.6 cm/s for O_2_ and CO_2_, respectively (Figure 6A,B). The calculated ΔG profiles indicate the highest accumulation of O_2_ and CO_2_ to be in the midplane of the membrane, corresponding to free energy minima ($\Delta G_{\min}$) of −2 kcal/mol for O_2_ and −1 kcal/mol for CO_2_ with respect to the bulk solution. The free energy maxima ($\Delta G_{\max}$) of ∼0.5 kcal/mol, indicating the lowest occupancy or solubility regions of the gas molecules, are located in the headgroup regions. Thus, according to the ΔG profiles, the free-energy barriers of diffusing from the center of the membrane to the aqueous solution ($\Delta G^{\mathrm{cen}\text{-}\mathrm{bulk}}$) are 2.5 kcal/mol for O_2_ and 1.25 kcal/mol for CO_2_ (Figure 7A,C).

For the simulated bilayers with 50 or 65 CHL mole%, the last 250 ns of the 325-ns simulations were used in $p_{d,mem}$ and ΔG calculations. O_2_ and CO_2_ remain accumulated in the membrane relative to the aqueous solution, and $\Delta G_{\min}$ also remains located in the midplane of the membrane. The incorporation of CHL molecules in the membrane decreases the occupancy of the gas molecules in the tail regions, as indicated by higher $\Delta G_{\mathrm{tail}}$ values, peaked at z=±10 Å (Figure 7A). In the pure POPC bilayers, the ΔG value drastically decreases as the gas molecule diffuses from the head group region towards the center of the membrane. This decrease in membrane solubility, however, does not decrease O_2_ permeability. At 50 CHL mole%, $\Delta G_{\mathrm{tail}}$ plateaus at ∼0 kcal/mol, so $\Delta G^{\mathrm{cen}\text{-}\mathrm{bulk}}$ for O_2_ is ∼0.5 kcal/mol lower than in the pure POPC bilayer, which results in a higher $p_{d,\mathrm{mem}}$ value of 16 cm/s. At 65 mole%, $\Delta G_{\mathrm{tail}}$ increases to 0.5 kcal/mol, forming new local $\Delta G_{\min}$ at ±18 Å. Still, $\Delta G^{\mathrm{cen}\text{-}\mathrm{bulk}}$ is about the same as that in the pure POPC bilayer (Figure 6A), as reflected by a $p_{d,\mathrm{mem}}$ value of 10 cm/s, which corresponds to a 16 % decrease.

In contrast to O_2_, the presence of high CHL contents decreases CO_2_ permeability (Figure 6B). The calculated $p_{d,\mathrm{mem}}$ values are ∼10 cm/s at 50 mole%, and ∼6 cm/s at 65 mole%. $\Delta G_{\mathrm{tail}}$ are ∼0.25 kcal/mol at 50 mole%, and ∼1 kcal/mol at 65 mole%, becoming the new global $\Delta G_{\max}$ (Figure 7C).

#### 3.2.2. Special Cases of Pure PSM and Binary PSM:CHL Bilayers

For the PSM bilayers with 0 and 50 CHL mole%, the last 400 ns of the 525-ns simulation trajectories were used to calculate the $p_{d,\mathrm{mem}}$ and ΔG profiles. The solubility of the gas molecules remains highest at the midplane of the membrane. In the pure PSM bilayers, no local $\Delta G_{\max}$ is formed in the tail regions despite substantial alterations of membrane structure and lipid dynamics compared with the pure POPC bilayers. $\Delta G^{\mathrm{cen}\text{-}\mathrm{bulk}}$ remains ∼2.5 kcal/mol for O_2_, and 1.5 kcal/mol for CO_2_, as in the pure POPC bilayers (Figure 7B,D). However, gas permeability in the pure PSM bilayers becomes appreciably smaller (by a factor of 10) than in the pure POPC and binary POPC:CHL bilayers. The calculated $p_{d,\mathrm{mem}}$ values are ∼1 cm/s for both O_2_ and CO_2_ (Figure 6A,B).

At 50 CHL mole%, the calculated $p_{d,\mathrm{mem}}$ for O_2_ is ∼2 cm/s, which is two times higher than CHL-free membranes. It is still at least five times lower than any of the simulated POPC bilayers. With the $\Delta G_{\max}$ at the tail regions, $\Delta G^{\mathrm{cen}\text{-}\mathrm{bulk}}$ is ∼3 kcal/mol (Figure 7B). For CO_2_, the calculated $p_{d,\mathrm{mem}}$ value is ∼1 cm/s (Figure 6B), while $\Delta G^{\mathrm{cen}\text{-}\mathrm{bulk}}$ increases to ∼2.75 kcal/mol (Figure 7C).

#### 3.2.3. POPC:PSM:CHL and POPC:DPPC:CHL Bilayers

POPC contains an unsaturated fatty chain and a saturated one, whereas PSM contains a saturated fatty chain and a sphingosine chain in the trans conformer. The saturation of the lipid tail could modulate the permeation of gas molecules, as the pure PSM bilayers appear to resemble a gel phase under the simulated temperature of 310 K. We explored this potential effect by simulating CO_2_ dynamics in a new set of membrane systems constituted of either POPC and PSM or POPC and DPPC (fully saturated). This new set included 50:50:0, 33:33:33, and 25:25:50 POPC:PSM:CHL, and 50:50:0 and 33:33:33 POPC:DPPC:CHL bilayers.

Similar to the other simulated systems, the highest solubility region for the gas molecules is the center of the membrane. Like the simulated CHL-free bilayers, no local $\Delta G_{\max}$ is apparent in the tail regions. $p_{d,\mathrm{mem}}$ are 8.4 cm/s and 14.3 cm/s in the 50:50 POPC:PSM and POPC:DPPC bilayers, respectively (Figure 6B,C), in correlation with the differences in lipid order and packing density. At 33 and 50 CHL mole%, CO_2_ occupancy is reduced in the tail region but to a lesser degree than the 50:50 PSM:CHL bilayer. CO_2_ permeability was also higher than in the pure PSM and 50:50 PSM:CHL bilayers. $p_{d,\mathrm{mem}}$ in the 33:33:33 POPC:PSM:CHL and POPC:DPPC:CHL bilayer was 6.5 cm/s and 7 cm/s, respectively (Figure 6B,C), in correlation with lipid occupancy profiles (Figure 2H,K). The value in the 25:25:50 POPC:PSM:CHL bilayer was 4.3 cm/s.

We also simulated CO_2_ diffusion in a DPPC bilayer with 50 CHL mole% and analyzed its last 400 ns trajectory for $p_{d,\mathrm{mem}}$ and ΔG calculations. The calculated $p_{d,\mathrm{mem}}$ value was ∼3 cm/s (Figure 6C), which is lower than those in the simulated POPC-containing bilayers but higher than the pure PSM and 50:50 PSM:CHL bilayers. $\Delta G^{\mathrm{cen}\text{-}\mathrm{bulk}}$ was ∼2.25 kcal/mol, which is 0.5 kcal/mol lower than in the 50:50 PSM:CHL bilayer (Figure 7E).

### 3.3. Diffusivity of Gas Molecules

Following the inhomogeneous solubility diffusion model [3,77,78,79], the membrane permeability of a gas molecule is modulated by its solubility or partitioning in the membrane, its diffusivity, and the membrane thickness. $p_{d,\mathrm{mem}}$ is expressed as

$$\frac{1}{p_{d,\mathrm{mem}}}=\int_{-d}^{+d}\frac{dz}{D\left(z\right)e^{-\Delta G(z)/RT}},$$

where ΔG(z) is the local partitioning free energy and D(z) is the local translational diffusion coefficient in membrane segment (d*z*). +d and −d corresponds to the upper and lower membrane parts for which diffusivity is calculated, respectively; the distance resembles the membrane height, denoted as Δz in Table 2. The integral of exp(−ΔG(z)/RT) is the partitioning or solubility coefficient of a gas molecule with respect to the aqueous solution and can be calculated from the partitioning ΔG profiles (Figure 7). With the calculated $p_{d,\mathrm{mem}}$, the overall diffusion coefficients of a gas molecule in the membrane ($D_{\mathrm{app}}$) can be approximated to

$$D_{app}=p_{d,\mathrm{mem}}\int_{-d}^{+d}\frac{dz}{e^{-\Delta G(z)/RT}}.$$

**Figure 8:**
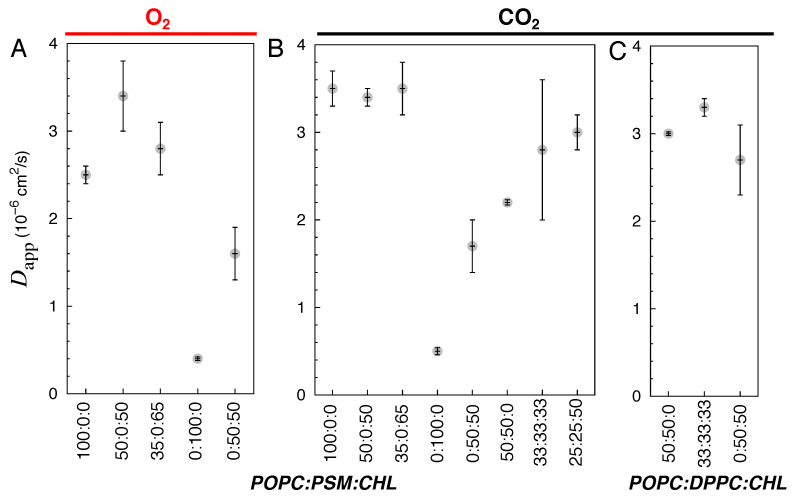
Approximated overall diffusion coefficients ($D_{\mathrm{app}}$) of O_2_ (**A**) and CO_2_ in POPC:PSM:CHL membranes (**B**) and of CO_2_ in POPC:DPPC:CHL membranes (**C**).

For all of the simulated POPC containing bilayers (i.e., pure POPC, POPC:CHL binary, and POPC:PSM:CHL and POPC:DPPC:CHL ternary bilayers), as well as for 50:50 DPPC:CHL, the decrease in the permeability of O_2_ and CO_2_ resulted from a decrease in their membrane solubility. The estimated $D_{\mathrm{app}}$ values for O_2_ and CO_2_ in these membranes are similar, ranging from $2\times 10^{-6}$ to $3.5\times 10^{-6}$ cm^2^/s (Figure 8), which is about 10 times slower than their diffusion in water ($∼3\times 10^{-5}$ cm^2^/s).

In the 50:50 PSM:CHL bilayers, $D_{\mathrm{app}}$ are $∼1.5\times 10^{-6}$ cm^2^/s (Figure 8A,B). Gas diffusion is even slower in the pure PSM bilayers with $D_{\mathrm{app}}$ values of $∼5\times 10^{-7}$ cm^2^/s (Figure 8A,B), which is at least 4 times slower than those in the simulated POPC-containing bilayers.

## 4. Discussion

**Figure 9:**
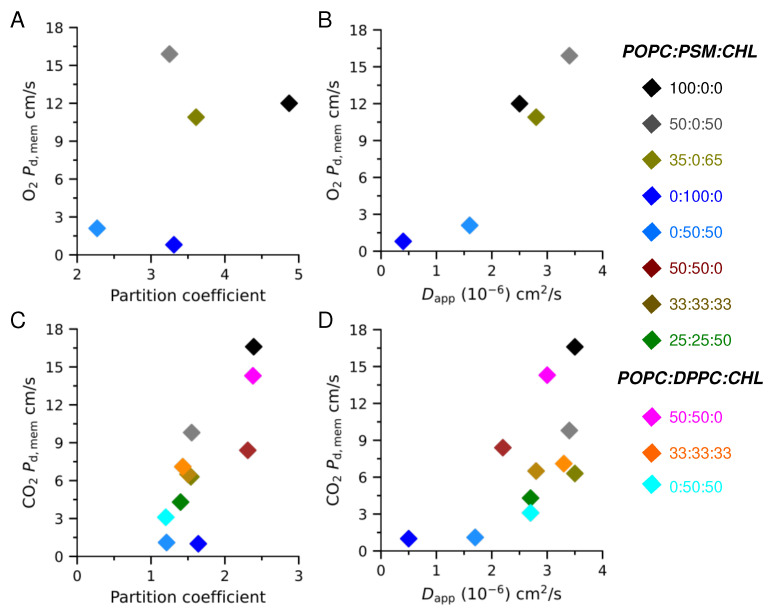
O_2_ and CO_2_ permeabilities calculated for different membranes plotted against either solubilities (**A**,**C**) or the overall diffusion coefficients ($D_{\mathrm{app}}$) of the gas in the membrane (**B**,**D**).

The present MD study examines the extent to which changes in the lipid compositions, such as the CHL and SM contents and the degree of lipid saturation, influence the permeability of bioactive O_2_ and CO_2_ gases across the membrane. Our results show that increasing CHL content results in an increase in lipid order, packing density, and membrane thickness, in correlation with a reduced gas solubility in the membrane (Figure 6, Figure 8 and Figure 9).

The degree of lipid saturation (i.e., presence of POPC, PSM, and/or DPPC) also contributes to the rate of gas permeation. Among the simulated bilayers, gas permeability is lowest in a pure PSM bilayer ($p_{d,\mathrm{mem}}∼1$ cm/s), and the incorporation of monosaturated POPC lipids increases the permeability by several folds.

We note that our simulation used a temperature of 310 K (body temperature). According to an X-ray diffraction study, the ripple-fluid phase transition temperature of the PSM bilayer is at 314 K [80]. This explains the observed undulated membrane structure at the simulated temperature (Figure 3). This also results in the dynamics of lipid molecules in the pure PSM bilayer being slower than those in the simulated POPC or CHL bilayers, which are either in a liquid-ordered or liquid-disordered phase or coexist in both phases [44,81]. Low membrane fluidity, together with tight packing of PSM lipids, rationalizes the appreciably lower calculated $p_{d,\mathrm{mem}}$ and $D_{\mathrm{app}}$ values for O_2_ and CO_2_ in the pure PSM bilayers. The apparently larger variations in the ΔG profiles in the PSM bilayer than in the CHL-free POPC, POPC:PSM, and POPC:DPPC bilayers (Figure 7) are most likely due to the coexistence of solid and liquid lipid domains in the ripple phase.

We also show that the presence of CHL in the membrane limits the permeability of CO_2_ more significantly than O_2_ (Figure 6A,B). These findings agree with a recent MD study [57], reporting only 23% reduction in O_2_ permeability at 62.5 CHL mole%. CHL partitioning in the membrane was also found to strengthen the hydrophobic lipid-lipid contacts [55], thereby tightening the membrane. This may make CO_2_, which has a larger volume, more difficult to diffuse than O_2_. This observation also explains the lower $p_{d,\mathrm{mem}}$ value for CO_2_ than O_2_, as CO_2_ shows a greater decrease in its solubility in the lipid-tail region occupied by CHL molecules (Figure 7A,C).

Comparison of the trends in Figure 6 and Figure 8 provides additional information about the differences between O_2_ and CO_2_ transports. Panels A of these two figures show very similar patterns for O_2_, suggesting that in this case the solubilities must be similar for this gas in different lipid bilayers, a prediction consistent with our free energy calculations showing the absence of a major barrier for this gas in different bilayers. For CO_2_, however, the patterns of $D_{\mathrm{app}}$ and $p_{d,\mathrm{mem}}$ are quite different, suggesting that in this case the solubilities are major factors in determining the overall permeability. Since the pure POPC and other simulated CHL bilayers have similar $D_{\mathrm{app}}$ values (Figure 8), gas permeation through fluid bilayers still follows the Overton rule, which predicts their permeability from their solubility [82].

In all of our simulated bilayers, gas concentration is highest in the midplane of the membrane. ΔG barriers for the entry of gas molecules from the aqueous solution into the membrane is negligibly small. It is the diffusion of gas molecules from the midplane to the solution that constitutes the main barrier to their permeation. These features could play vital roles in a living cell by creating a local concentration gradient of gases that are substrates of many biochemical reactions taking place within the membrane. A notable example is the catalysis of O_2_ to water by cytochrome *c* oxidases of which the entrance of the O_2_ access pathway is located near the center of the membrane [83].

Many aspects potentially controlling membrane permeability remain to be explored, and this study focuses only on neutral phospholipids (POPC, DPPC, and PSM) and CHL. Realistic biological membranes also contain anionic and more complex lipids, as well as integral and peripheral proteins and adhesion proteins. These other components, especially proteins, are structurally more condensed and less dynamic than lipids, so their presence could add significant barriers for gas molecules to penetrate. In such conditions, membrane channels, such as aquaporins, which contain high-affinity, always-open pathways for gases [84,85], may be important for maintaining cellular homeostasis and reducing cellular toxicity.

## Figures and Tables

**Table 1:** Lipid bilayer systems simulated in this study.

Lipid Ratio	Lipid Numbers	System Size (Atoms)
**POPC:PSM:CHL**		
100:0:0	294/0/0	77,406
50:0:50	186/0/186	79,413
35:0:65	154/0/286	85,207
0:100:0	0/362/0	80,823
0:50:50	0/210/210	84,098
50:50:0	162/162/0	83,253
33:33:33	124/124/124	81,324
25:25:50	100/100/200	82,599
**POPC:DPPC:CHL**		
50:50:0	154/154/0	78,708
0:50:50	0/196/196	79,346
33:33:33	118/118/118	78,997

**Table 2:** Calculated membrane permeability of O_2_ and CO_2_.

	δt	<[gas^bulk^]>	<*Area*^*xy*^>	Δz	*p*_*d*,*mem*_	*D*_*app*_
	(ns)	(mM)	(Å^2^)	(Å)	(cm/s)	(10^−6^ cm^2^/s)
**POPC:PSM:CHL**	** O_2_**					
100:0:0	175	54	9486	44	12 ± 0.4	2.5 ± 0.1
50:0:50	250	86	8022	46	15.9 ± 1.8	3.4 ± 0.4
35:0:65	250	77	8967	44	10.9 ± 1.0	2.8 ± 0.3
0:100:0	400	76	9245	48	0.8 ± 0.1	0.4 ± 0.0
0:50:50	400	115	8346	50	2.1 ± 0.4	1.6 ± 0.3
**POPC:PSM:CHL**	** CO_2_**					
100:0:0	175	125	9545	44	16.6 ± 0.8	3.5 ± 0.2
50:0:50	250	182	8017	46	9.8 ± 0.3	3.4 ± 0.1
35:0:65	250	173	8958	44	6.3 ± 0.6	3.5 ± 0.3
0:100:0	400	165	9154	48	1.0 ± 0.1	0.5 ± 0.0
0:50:50	400	223	8342	50	1.1 ± 0.2	1.7 ± 0.3
50:50:0	250	117	9473	46	8.4 ± 0.1	2.2 ± 0.0
33:33:33	250	178	8162	50	6.5 ± 1.8	2.8 ± 0.8
25:25:50	250	185	8206	50	4.3 ± 0.3	2.7 ± 0.4
**POPC:DPPC:CHL**	** CO_2_**					
50:50:0	250	122	9572	44	14.3 ± 0.1	3.0 ± 0.0
33:33:33	250	198	7891	48	7.1 ± 1.1	3.3 ± 0.1
0:50:50	400	241	7828	50	3.1 ± 0.1	2.7 ± 0.4

## Data Availability

The trajectory files are available upon request.
